# Non-Adherence to Anti-Retroviral Therapy Among Adult People Living with HIV in Ethiopia: Systematic Review and Meta-Analysis

**DOI:** 10.1007/s10461-023-04252-4

**Published:** 2023-12-29

**Authors:** Tigabu Munye Aytenew, Solomon Demis, Binyam Minuye Birhane, Worku Necho Asferie, Amare Simegn, Gedefaye Nibret, Amare Kassaw, Sintayehu Asnakew, Yohannes Tesfahun, Henock Andualem, Berihun Bantie, Gebrie Kassaw, Demewoz Kefale, Shegaw Zeleke

**Affiliations:** 1 Department of Nursing, College of Health Sciences, Debre Tabor University, Debre Tabor, Ethiopia; 2 Department of Maternity and Neonatal Nursing, College of Health Sciences, Debre Tabor University, Debre Tabor, Ethiopia; 3 School of Public Health, University of Technology Sydney, Sydney, NSW Australia; 4 Department of Reproductive Health, College of Health Sciences, Debre Tabor University, Debre Tabor, Ethiopia; 5 Department of Pediatrics and Child health Nursing, College of Health Sciences, Debre Tabor University, Debre Tabor, Ethiopia; 6 Department of Psychiatry, College of Health Sciences, Debre Tabor University, Debre Tabor, Ethiopia; 7 Department of Emergency and Critical Care Nursing, College of Health Sciences, Debre Tabor University, Debre Tabor, Ethiopia; 8 Department of Medical Laboratory, College of Health Sciences, Debre Tabor University, Debre Tabor, Ethiopia

**Keywords:** Non-adherence to ART, Predictors, Ethiopia, Meta-analysis

## Abstract

Human immunodeficiency virus remains a global public health problem. Despite efforts to determine the prevalence of non-adherence to ART and its predictors in Ethiopia, various primary studies presented inconsistent findings. Therefore, this review aimed to determine the pooled prevalence of non-adherence to ART and identify its predictors. We have searched PubMed, Google Scholar and Web of Science databases extensively for all available studies. A weighted inverse-variance random-effects model was used to compute the overall non-adherence to ART. The pooled prevalence of non-adherence to ART was 20.68% (95% CI: 17.74, 23.61); I^2^ = 98.40%; p < 0.001). Educational level of primary school and lower [AOR = 3.5, 95%CI: 1.7, 7.4], taking co-medications [AOR = 0.45, 95%CI: 0.35, 0.59], not using memory aids [AOR = 0.30, 95%CI: 0.13, 0.71], depression [AOR = 2.0, 95%CI: 1.05, 3.79], comorbidity [AOR = 2.12, 95%CI: 1.16, 3.09), under-nutrition [AOR = 2.02, 95%CI: 1.20, 3.43], not believing on ART can control HIV [AOR = 2.31, 95%CI: 1.92, 2.77], lack of access to health facilities [AOR = 3.86, 95%CI: 1.10, 13.51] and taking ART pills uncomfortably while others looking [AOR = 5.21, 95%CI: 2.56, 10.53] were significantly associated with non-adherence to anti-retroviral therapy. The overall pooled prevalence of non-adherence to ART was considerably high in Ethiopia. Educational status, taking co-medications, not using memory aids, depression, comorbidity, under nutrition, not believing on anti-retroviral therapy controls HIV, lack of access to health facilities and taking ART pills uncomfortably were independent predictors of non-adherence to ART in Ethiopia. Therefore, healthcare providers, adherence counselors and supporters should detect non-adherence behaviors and patients’ difficulties with ART early, and provide intensive counseling to promote adherence.

**Supplementary Information:**

The online version contains supplementary material available at 10.1007/s10461-023-04252-4.

## Introduction

Human immunodeficiency virus/acquired immunodeficiency syndrome (HIV/AIDS) remains a major global public health problem [1–10]. As of 2021, it is estimated that 38.4 million people across the globe are living with HIV. In the same year, about 1.5 million new cases were recorded, and there were 650,000 deaths related to HIV/AIDS [11, 12]. Of this, the highest burden (60–90%) was accounted for developing countries including Ethiopia [2, 13, 14]. According to the Ethiopian Demographic and Health Survey (EDHS), 2016 report, the prevalence of HIV among the adult population was estimated to be 1.18% [15]. The World Health Organization (WHO) global disease burden, 2016 also reported that 768,040 people were living with HIV, 39,140 new cases, and 28,650 HIV/AIDS-related deaths in Ethiopia [16].

HIV/AIDS not only upset individuals’ health, but also influences the families, communities, sectors and the development of the nation [14, 17–19]. The introduction of anti-retroviral therapy (ART) was a crucial turning point in the history of HIV infection, and the gradual evolution of the infection into a chronic and non-fatal condition [20]. The primary goal of ART is to achieve and sustain viral suppression [20–22]. The initial challenge in combating HIV/AIDS epidemic was access to anti-retroviral (ARV) drugs [2, 23, 24].

However, increasing the production and decreasing the cost of ARV drugs intensively have facilitated the scaling-up of ART programs worldwide [23, 24]. The Joint United Nations Programme on HIV/AIDS set the 95-95-95 targets in order to combat the spread of HIV. By the year 2030, it is aimed that 95% of all people living with HIV (PLWH) will be aware of their HIV status. Additionally, 95% of those who are diagnosed with HIV will receive antiretroviral therapy [25, 26].

ART is recommended for all patients irrespective of their CD4 cell count, WHO staging and clinical status (test and treat strategy) [27, 28]. Early initiation and strict adherence to ART are important in the reduction of the progression of the virus through suppression of viral load and increase the level of cluster of differentiation 4 (CD4) cells count. This improves the survival and quality of life of the patient, increases productivity and decreases the incidence of opportunistic infections [5, 29, 30]. It is also believed that ART prevents the chance of HIV transmission by suppressing the viral load in infected individuals [31–33].

Adherence to ART is the patient’s ability to follow treatment plan by taking the correct dose of medications (≥ 95% of the prescribed doses) at prescribed time, frequencies (schedule) and following dietary instructions [9, 18, 34, 35]. It is strongly correlated with suppression of the disease and reduction of morbidity and mortality rates among people living with HIV [36–38], and the clinical outcomes of ART mainly depends on the adherence of patients to ART [19].

However, it often poses a special challenge and requires commitment from both the patient and the healthcare team [1, 39]. Non-adherence to ART is a major reason for treatment failure [35, 40, 41]. Ethiopia has implemented various strategies to enhance adherence to ART. These include transforming a fee-based ART program into a free one in 2005, decentralizing services to lower level health facilities and private hospitals and offering capacity building for service providers on counseling aspects [6, 42]. However, ensuring adherence to ART remains a major challenge in the country [6, 43].

Despite many efforts to determine the prevalence of non-adherence to ART and its predictors among adult people living with HIV in Ethiopia, various primary studies presented inconsistent findings and showed epidemiological variations ranging from 3% [2] to 61% [44]. Therefore, this review aimed to determine the pooled prevalence of non-adherence to ART and identify its predictors.

## Methods

### Reporting and Registration Protocol

The Preferred Reporting Items for Systematic Reviews and Meta-Analyses (PRISMA) checklist [45] was used to report the results of this systematic review and meta-analysis (Supplemental Table 1). The review protocol was registered with Prospero database: (PROSPERO, 2023: CRD42023429516).

### Databases and Search Strategy

We have extensively searched PubMed, Google Scholar, Web of Science databases for all available primary studies reporting non-adherence to ART and its predictors in Ethiopia using the following search terms and phrases: (ʺNon-adherenceʺ [MeSH term] OR ʺAdherenceʺ [MeSH term] OR ʺPrevalenceʺ [MeSH term]) AND (ʺAnti-retroviral therapyʺ [MeSH term]) AND (ʺPredictorsʺ [MeSH term] OR ʺAssociated factorsʺ [MeSH term] OR ʺRisk factorsʺ [MeSH term] OR ʺDeterminantsʺ [MeSH term]) AND ʺEthiopiaʺ. The search string was developed using ʺANDʺ and ʺORʺ Boolean Operators. Moreover, a manual search of the reference lists of included studies was also performed. The searched studies were published between 2007 and 2023 in Ethiopia and published in English.

### Eligibility Criteria

All observational (cross-sectional, case-control and retrospective cohort) studies that were conducted among adult (age ≥ 18 years old) people living with HIV and which have reported the prevalence of non-adherence to ART and/or at least one associated factor of non-adherence to ART and written in English were included in the review. However, citations without abstracts, full texts, anonymous reports, editorials, systematic reviews and meta-analyses and qualitative studies were excluded from the review.

### Study Selection

All the retrieved studies were exported to EndNote version 7 reference manager and the duplicated studies were removed. Initially, two independent reviewers (TM and SD) screened the titles and abstracts, followed by the full-text reviews to determine the eligibility of each study. The disagreement between the two reviews was solved through discussion.

### Data Extraction

Two independent reviewers (TM and SZ) have extracted the data using a structured Microsoft excel data extraction form. Whenever variations were observed in the extracted data, the phase was repeated. While the discrepancies between the data extractors were continued, the third reviewer (AS) was involved. The name of the first author and year of publication, region, study area, study design, sample size, response rate and effect size of the included primary studies were extracted.

### Primary Outcome Measure of Interest

The primary outcome of interest was prevalence of non-adherence to ART among adult people living with HIV in Ethiopia, which was determined by dividing the total number of non-adherents by the total number of study participants.

### Operational Definitions of Variables

Non-adherence to ART is the condition of missing doses completely, not following the information given by the health care providers and taking drugs inappropriately [9, 18, 34, 35].

### Data Analysis

STATA version 17 statistical software was used to analyze all the statistical analyses. A weighted inverse-variance random-effects model [46] was used to compute the overall non-adherence to ART and determine the impact of its predictors.

The presence of publication bias was checked by observing the symmetry of the funnel plot, and Egger’s test with a p-value of < 0.05 was also employed to determine a significant publication bias [47]. The percentage of total variation across studies due to heterogeneity was assessed using I^2^ statistics [48]. The values of I^2^ 25,50 and 75% represented low, moderate and high heterogeneity respectively [48].

A p-value of I^2^ statistic < 0.05 was used to declare a significant heterogeneity [49, 50]. To identify the influence of a single study on the overall meta-analysis, sensitivity analysis was performed. A forest plot was used to estimate the effect of independent factors on the outcome variable and a measure of association at 95%CI was reported. The Odds Ratio (OR) was the most frequently reported measure of association in the eligible primary studies. When the included primary studies provided Risk Ratio results rather than ORs, we used Epi-Info along with descriptive statistics to convert these statistics to ORs.

To estimate the pooled OR effect, either a fixed-effects or a random-effects model is used. A fixed-effects model is used if all the included studies used comparable methodology and were from identical populations, whereas a random-effects model is used when the included studies used different methodologies and sampled from different populations. In our review, the included primary studies used different methodologies and drawn from several independent populations. Thus, a random-effects model was used for this study.

## Results

### Search Results

The search strategy retrieved a total of 2137 studies from PubMed (n = 1156), Google Scholar (n = 953), Web of Science (n = 16), manual search (n = 7) and University’s research repository online library (n = 5) studies. After carefully removing irrelevant studies based on their titles and abstracts (n = 1596) and duplicated studies (n = 112), a total of 429 studies were selected for full-text review.

**Fig. 1:**
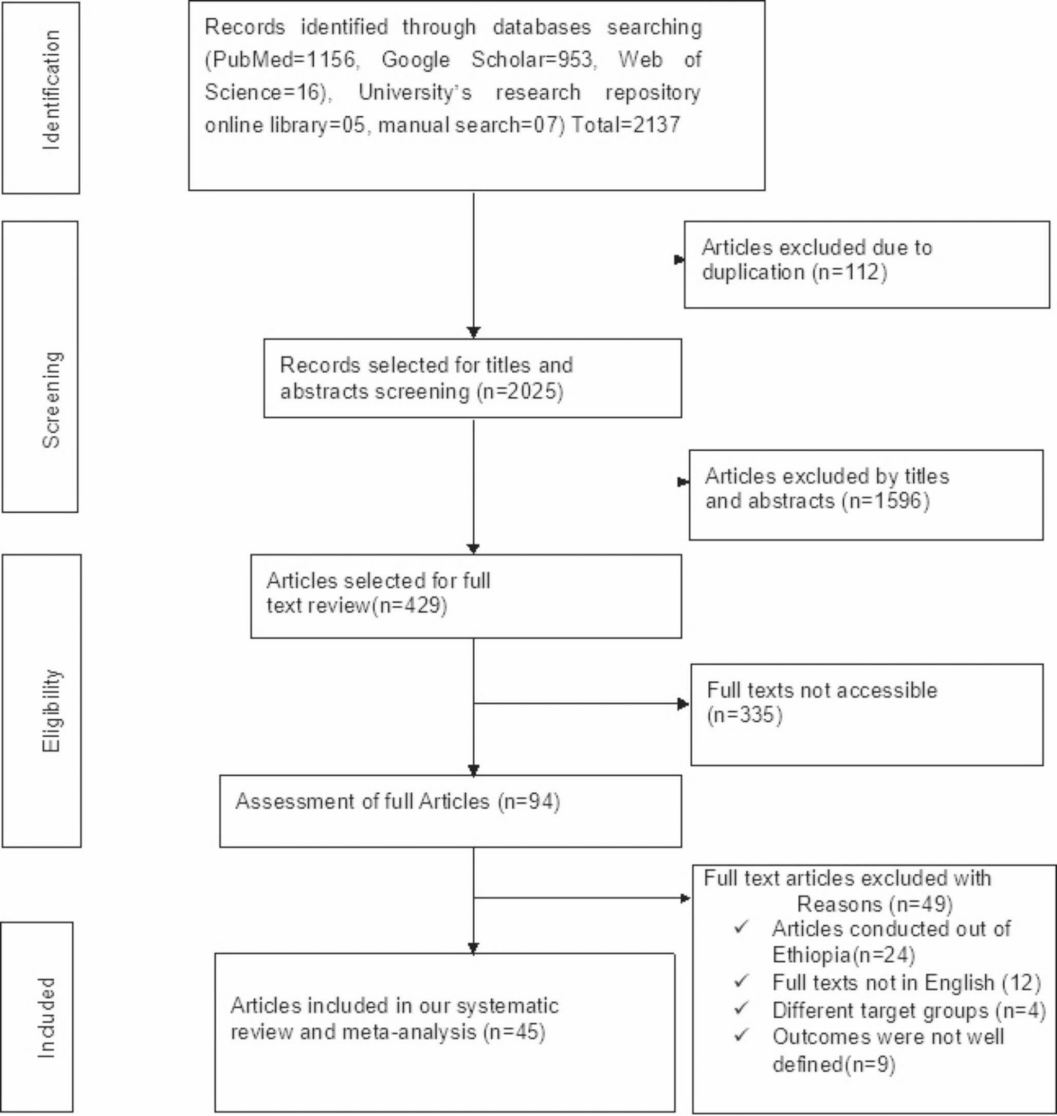
PRISMA flow chart showing the studies selection process, 2023

Afterward, full-text reviews were conducted, resulting in the removal of 335 studies due to lack of complete texts. Then, 94 studies were assessed for full articles review and 49 studies were excluded (their full texts not written in English, conducted outside of Ethiopia, different target groups and the outcomes not well defined). Finally, 45 studies were found relevant to determine the prevalence of non-adherence to ART and identify its predictors. We traced the PRISMA flow chart [51] to show the selection process from initially identified records to finally included studies (Fig. 1).

### Characteristics of the Included Studies

The forty-two studies [1–3, 5, 6, 8–10, 18, 19, 27, 29, 34, 43, 44, 52–78], two studies [79, 80] and one study [35] were conducted using cross-sectional, retrospective cohort and case-control studies respectively. Regarding geographical region, fourteen studies were conducted in Oromia [2, 3, 9, 18, 27, 44, 52, 57, 59, 64, 69, 76, 77, 79], eleven studies in Amhara [1, 6, 19, 56, 61, 66–68, 73, 75, 78], ten studies in Southern nations nationalities [5, 10, 34, 35, 43, 53, 55, 60, 62, 72], two studies in Addis Ababa [8, 44], two studies in Tigray [58, 80], one study in Oromia and Southern nations nationalities [54], one study in Harari and Dire Dawa [63] and one study was conducted in each Benishangul Gumuz [71], Somali [74], Harari [29], and Sidama region [65]. The total sample size of the included studies was 36,317, where the smallest sample size was 81 in Southern nations and nationalities and the largest sample size was also 19,525 in Tigray region health research institute. The prevalence of non-adherence to ART was obtained from forty-four included primary studies [1–3, 5, 6, 8–10, 18, 19, 27, 29, 34, 43, 44, 52–80], while the data regarding the predictors of non-adherence to ART were obtained from the thirty-five studies [1, 2, 5, 6, 8, 18, 19, 27, 29, 35, 44, 53–55, 57–61, 63–69, 71–74, 76–80], with a response rate ranges from 87 to 100% (Table 1).

**Table 1:** General characteristics of the included studies

ID	Author [Year]	Study area	Study design	Sample size	Prevalence (95% CI)	Quality
1.	Abera A et al. [2015]	Oromia	CS	221	36.19(29.9, 42.5)	Low risk
2.	Alagaw A et al. [2013]	SNNP	CS	357	25.6(21.1, 30.1)	Low risk
3.	Amberbir A et al. [2008]	Oromia	Cohort	400	5.7(3.4, 8.0)	Low risk
4.	Angelo AT et al. [2021]	SNNP	CS	329	16.7(12.7, 20.7)	Low risk
5.	Assefa N et al. [2021]	A. A	CS	422	61(56.4, 65.7)	Low risk
6.	Awel M et al. [2007]	Oromia	CS	459	17(13.6, 20.4)	Low risk
7.	Aychiluhm SB et al. [2021]	Amhara	CS	310	17.4(13.2, 21.6)	Low risk
8.	Beyene KA et al. [2009]	Oromia + SNNP	CS	422	6.9(4.5, 9.3)	Low risk
9.	Billoro BB et al. [2018]	SNNP	CS	265	43.8(37.8, 49.8)	Low risk
10.	Bitew BD et al. [2014]	SNNP	Case-control	462	Not applicable	Low risk
11.	Chaka TE et al. [2016]	Oromia	CS	1,631	3(2.2, 3.8)	Low risk
12.	Debito T et al. [2014]	SNNP	CS	341	21.4(17.1, 25.8)	Low risk
13.	Demas Z et al. [2022]	Oromia	CS	385	30.6(26.0, 35.2)	Low risk
14.	Demeke B et al. [2014]	Amhara	CS	130	10(4.8, 15.2)	Low risk
15.	Demessie R et al. [2014]	A. A	CS	350	20.9(16.6, 25.2)	Low risk
16.	Desta AA et al. [2020]	Tigray	Cohort	19,525	5.16(4.9, 5.5)	Low risk
17.	Dibaba D et al. [2021]	Oromia	CS	445	10.2(7.4, 13.0)	Low risk
18.	Ejigu M et al. [2020]	Oromia	CS	284	19(14.4, 23.6)	Low risk
19.	Ejigu SH *et a*l [2014]	Oromia	CS	271	21.1(16.2, 26.0)	Low risk
20.	Gebregziabher TT et al. [2020]	Tigray	CS	339	25.4(20.8, 30.0)	Low risk
21.	Hassen A et al. [2018]	Oromia	CS	352	26.4(21.8, 31.0)	Low risk
22.	Hebo SH et al. [2019]	SNNP	CS	355	32.23(27.4, 37.1)	Low risk
23.	Hussen HS et al. [2019]	SNNP	CS	391	32.23(27.6, 36.9)	Low risk
24.	Jima F et al. [2018]	Amhara	CS	160	14.4(9.0, 19.8)	Low risk
25.	Kassahun TB et al. [2018]	Oromia	CS	321	27.7(22.8, 32.6)	Low risk
26.	Koyra HC et al. [2018]	SNNP	CS	320	32(27.0, 37.1)	Low risk
27.	Letta S et al. [2016]	Harari + Dire D	CS	620	15(12.2, 17.8)	Low risk
28.	Markos E et al. [2008]	Sidama	CS	291	25.8()20.8, 30.8	Low risk
29.	Mengistie A et al. [2018]	Amhara	CS	352	5(2.7, 7.3)	Low risk
30.	Mitku H et al. [2013]	Harari	CS	239	13(8.7, 17.3)	Low risk
31.	Mitku AA et al. [2016]	Amhara	CS	224	10.3(6.3, 14.3)	Low risk
32.	Mohammed M et al. [2023]	Amhara	CS	394	33.2(28.6, 37.9)	Low risk
33.	Molla AA et al. [2018]	Amhara	CS	440	11.8(8.8, 14.8)	Low risk
34.	Negash E et al. [2016]	Oromia	CS	383	10.7(7.6, 13.8)	Low risk
35.	Nigusso FT et al. [2020]	B/Gumuz	CS	390	39.7(34.8, 44.6)	Low risk
36.	Reta H et al. [2017]	SNNP	CS	81	11(4.2, 17.8)	Low risk
37.	Rike M et al. [2021	SNNP	CS	370	19.7(15.7, 23.8)	Low risk
38.	Tadesse S et al. [2014	Amhara	CS	647	14.7(12.0, 17.4)	Low risk
39.	Tesfay S et al. [2022]	Somali	CS	373	23.1(18.8, 27.4)	Low risk
40.	Tessema B et al. [2010]	Amhara	CS	504	17.3(14.0, 20.6)	Low risk
41.	Tiyou A et al. [2010]	Oromia	CS	319	27.6(22.7, 32.5)	Low risk
42.	Tsega B et al. [2015]	Amhara	CS	351	19.1(15.0, 23.2)	Low risk
43.	Yadeta AD et al. [2016]	Oromia	CS	326	33.74(28.6, 38.9)	Low risk
44.	Zeleke AB et al. [2012]	Oromia	CS	334	6.29(3.7, 8.9)	Low risk
45.	Zewude SB et al. [2022]	Amhara	CS	432	18.5(14.8, 22.2)	Low risk

*Abbreviations*: *AA*, Addis Ababa; *B/Gumuz*, Benishangul Gumuz; *CS*, cross-sectional; *Dire D*, Dire Dawa;*SNNP*,Southern nations, nationalities and peoples

### Quality Appraisal of the Included Studies

Two independent reviewers (TM and SD) appraised the quality of the included studies ,and scored for the validity of results. The quality of each study was evaluated using the Joanna Briggs Institute (JBI) quality appraisal criteria [81]. Forty-two studies [1–3, 5, 6, 8–10, 18, 19, 27, 29, 34, 43, 44, 52–78], two studies [79, 80] and one study [35] were appraised using JBI checklist for cross-sectional, cohort and case-control studies respectively.

Thus, among the forty-two cross-sectional studies, thirty-four studies scored seven of eight questions, 87.5% (low risk), five studies scored six of eight questions, 75% (low risk), and the remaining three studies also scored five of eight questions, 62.5% (low risk). Likewise, among the two cohort studies, one study scored eight of ten questions, 80% (low risk), and the second study also scored seven of ten questions, 70% (low risk). Moreover, one case-control study was appraised and scored eight of ten questions (Supplemental Table 2).

Studies were deemed to be of low risk when they scored 50% or higher on the quality assessment indicators. After conducting a thorough quality appraisal, we determined that the primary studies included in our analysis displayed a high level of reliability in their methodological quality scores. The cross-sectional studies scored between 5 and 7 out of a total of 8 points, while the cohort and case-control studies scored between 7 and 8 out of a total of 10 points. Thus, all included studies [1–3, 5, 6, 8–10, 18, 19, 27, 29, 34, 35, 43, 44, 52–80] had high quality.

### Risk of Bias Assessment

The assessment tool [82] was used to assess the risk of bias. It consists of ten items that assess four areas of bias: internal validity and external validity. Items 1–4 evaluate selection bias, non-response bias and external validity. Items 5–10 assess measure bias, analysis-related bias, and internal validity. Accordingly, of the total of the forty-five included studies, thirty-nine studies scored eight of ten questions and the six studies also scored seven of ten questions. Studies were classified as ʺlow riskʺ if eight and above of ten questions received a ʺYesʺ, as ʺmoderate riskʺ if six to seven of ten questions received a ʺYesʺ and as ʺhigh riskʺ if five or lower of ten questions received a ʺYesʺ. Therefore, all included studies [1–3, 5, 6, 8–10, 18, 19, 27, 29, 34, 35, 43, 44, 52–80] had low risk of bias (high quality) (Supplemental Table 3).

### Meta-Analysis

#### Non-Adherence to Anti-Retroviral Therapy

**Fig. 2:**
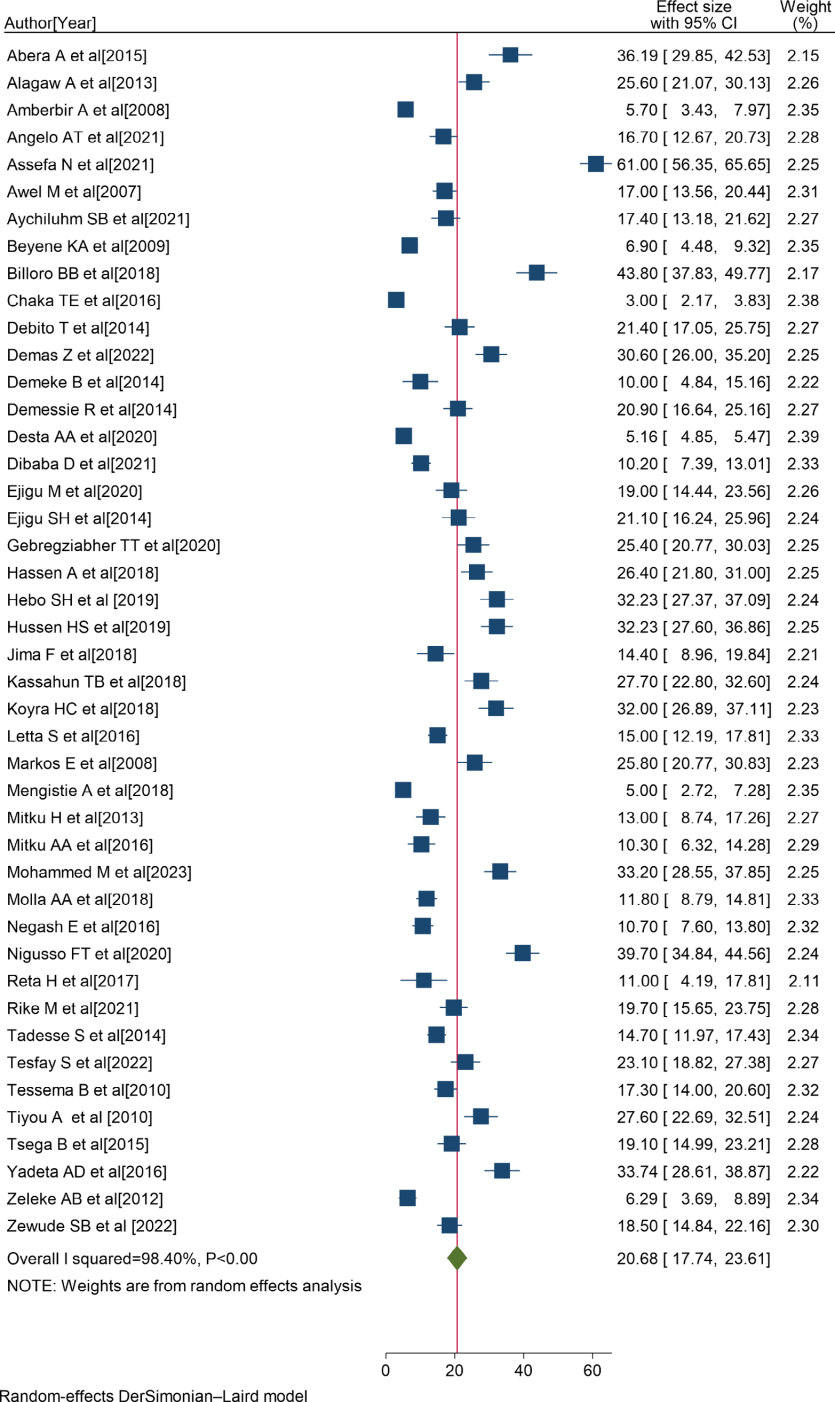
Forest plot showing the pooled non-adherence to ART in Ethiopia, 2023

Consequently, 45 eligible primary studies [1–3, 5, 6, 8–10, 18, 19, 27, 29, 34, 35, 43, 44, 52–80] were included in the final meta-analysis. In Ethiopia, the prevalence of non-adherence to ART ranges from 3% in Oromia [2] to 61% in Addis Ababa [44], and the overall pooled prevalence of non-adherence to ART was 20.68% (95% CI:17.74, 23.61); I^2^ = 98.40%; P < 0.001) (Fig. 2).

### Publication Bias

**Fig. 3:**
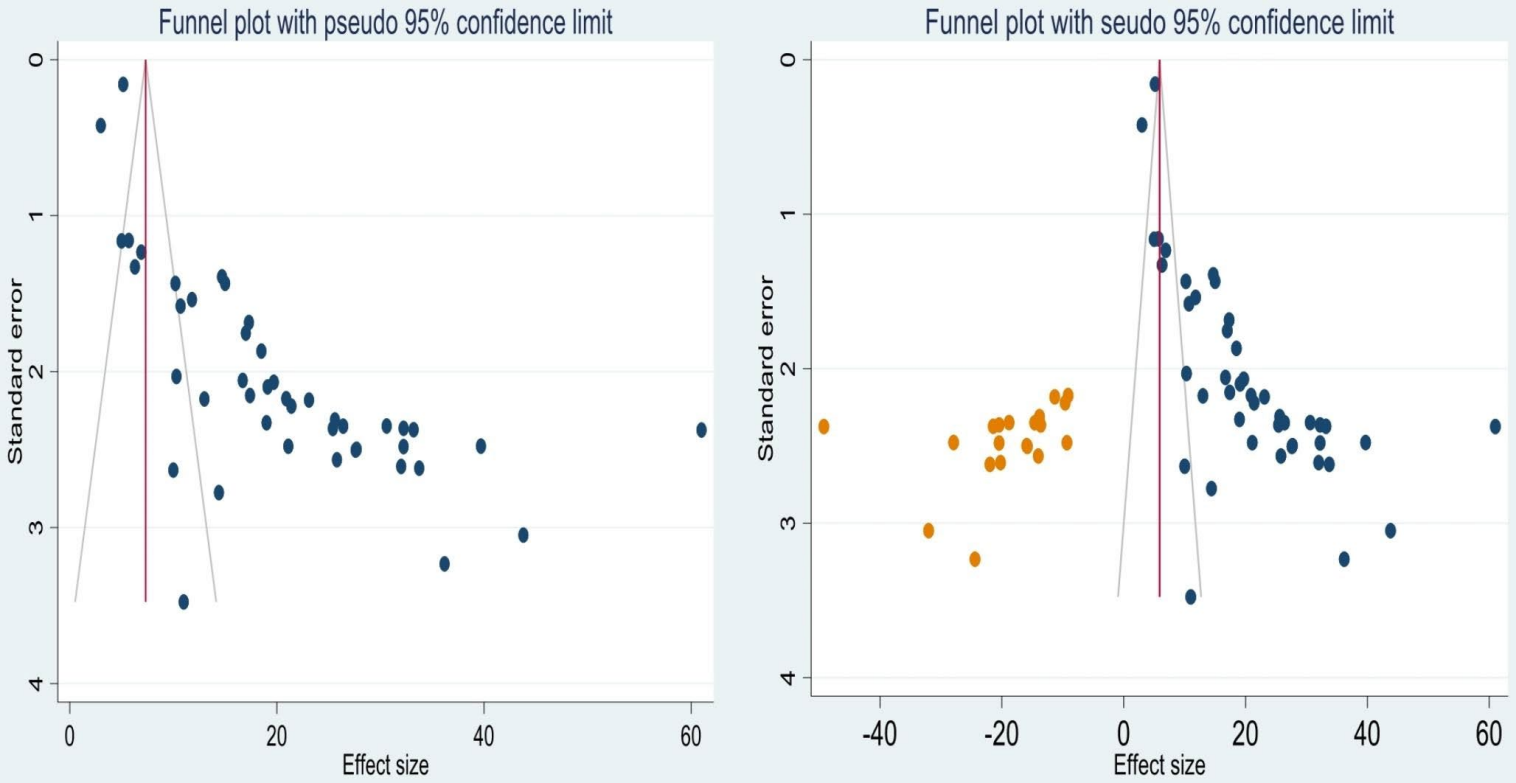
Funnel plot showing the publication bias of non-adherence to ART before adjustment **a** and after adjustment with trim and fill analysis **b** in Ethiopia, 2023

The asymmetry of the included primary studies on the funnel plot suggests the presence of publication bias (Fig. 3a), and the p-value of Egger’s regression test (P < 0.001) also indicated the presence of publication bias. Hence, we have done trim and fill analysis to manage the publication bias (Fig. 3b).

### Investigation of Heterogeneity

The percentage of I^2^ statistics of the forest plot indicates a marked heterogeneity among the included studies (I^2^ = 98.40%, P < 0.001) (Fig. 2). Hence, sensitivity analysis and sub-group analysis were performed to minimize the heterogeneity.

### Sensitivity Analysis

**Fig. 4:**
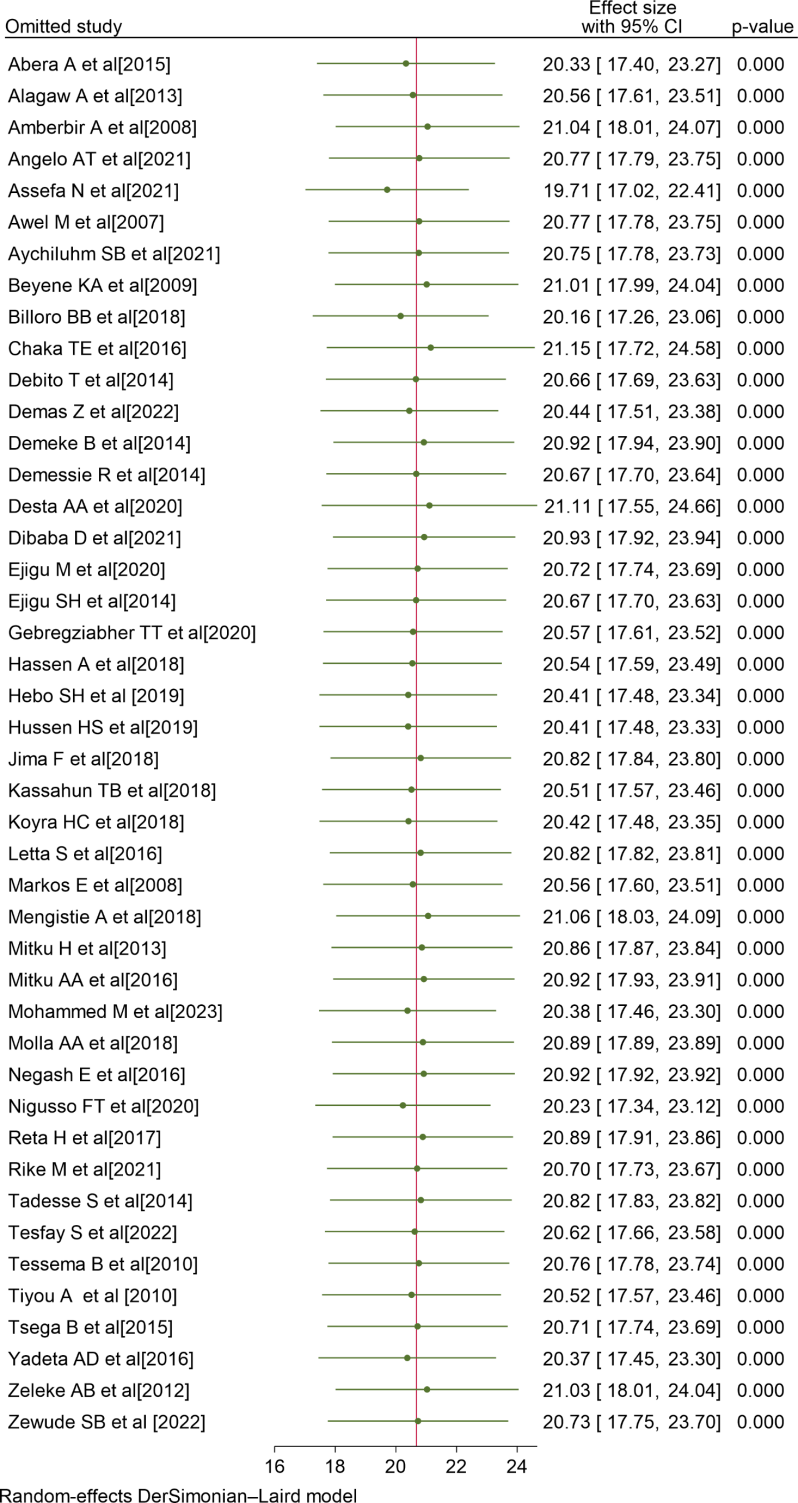
Sensitivity analysis of non-adherence to ART in Ethiopia, 2023

To determine the influence of a particular study on the overall meta-analysis, we conducted a sensitivity analysis. The forest plot showed that the estimate from a single study is closer to the combined estimate, which implied the absence of a single study effect on the overall pooled estimate. Thus, it has been demonstrated that a solitary study has no significant impact on the overall outcome of the meta-analysis (Fig. 4).

### Sub-group Analysis by the Sample Size

The sample size sub-group analysis showed the higher pooled prevalence of non-adherence to ART with <350 sample sizes [21.63, 95%CI: 17.10, 26.15, I^2^= 94.55%, P<0.001] followed by ≥350 sample sizes [19.94, 95%CI: 16.35, 23.52, I^2^= 98.70%, P<0.001] (Table 2).

**Table 2:** Subgroup analysis of non-adherence to ART in Ethiopia, 2023

Variables	Outcome	Subgroup	No. of studies	Model	Prevalence (95%CI)	I^2^	P-value
Sample size	ART non-adherence	≥ 350	25	Random	19.94(16.35, 23.52)	98.70%	0.00
		< 350	20	Random	21.63(17.10, 26.15)	94.55%	0.00
Study period	ART non-adherence	≥ 2018	23	Random	24.70(18.65, 30.74)	98.91%	0.00
		< 2018	22	Random	16.74(12.96, 20.53)	97.08%	0.00

### Sub-group analysis by the Study Period

The pooled prevalence of non-adherence to ART in studies conducted before the year 2018 was 16.74 [95%CI: 12.96, 20.53, I^2^ = 97.08%, P < 0.001], which was lower than the studies conducted in the year 2018 and later [24.70, 95% CI: 18.65, 30.74; I^2^ = 98.91%; P < 0.001] (Table 2). Based on the sub-group analyses, it appears that the heterogeneity of this study may be attributed to differences in sample sizes and study periods among the primary studies that were included.

### Predictors of Non-Adherence to ART

**Fig. 5:**
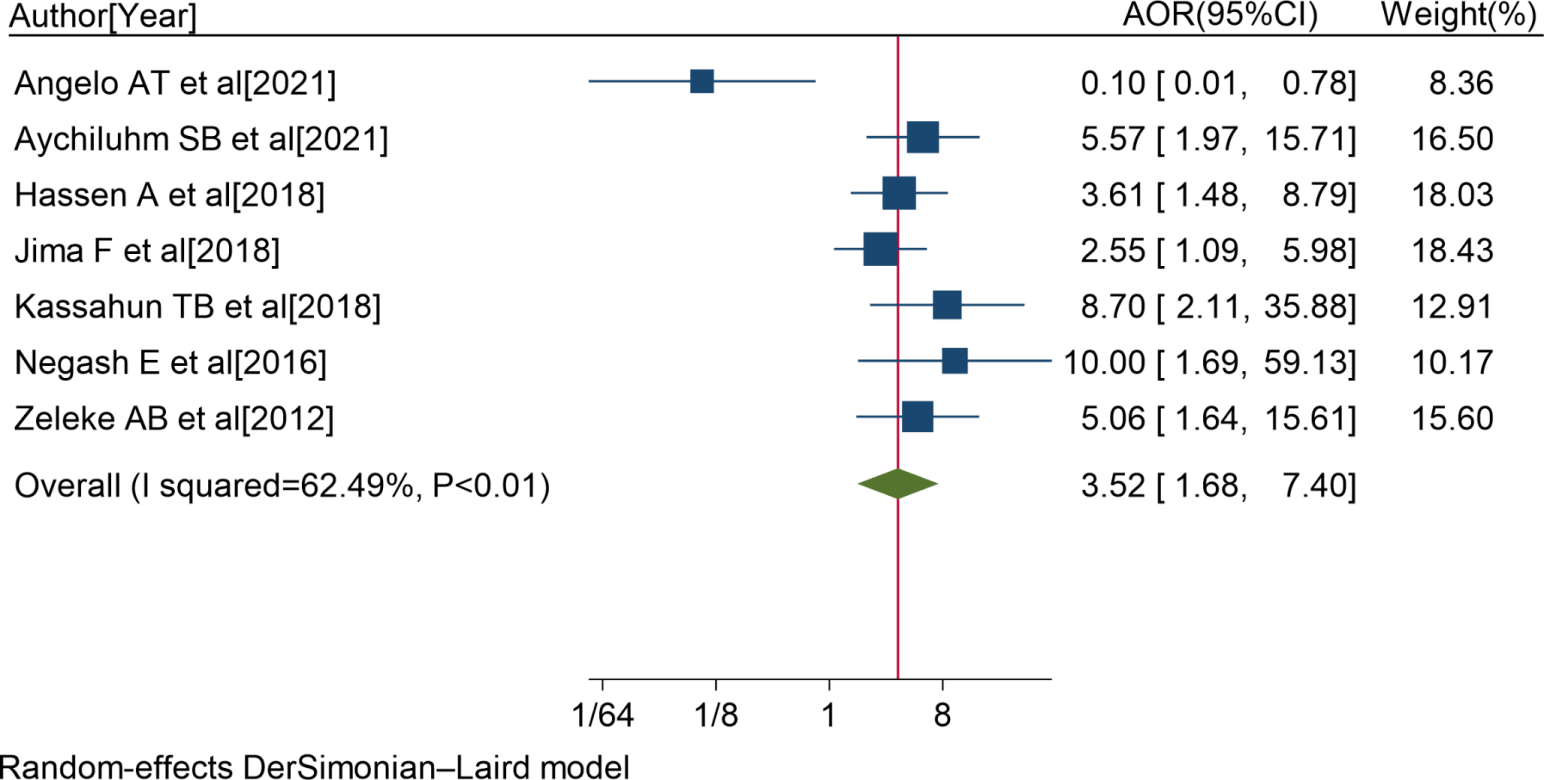
Forest plot of the adjusted odds ratios with corresponding 95% CIs of studies on the association of educational level of primary school and lower and non-adherence to ART. The midpoint and the length of each segment indicated an AOR and a 95% CI; and the diamond shape showed the combined AOR of all studies

In the review, seven studies [5, 19, 52, 54, 59, 61, 64] reported that educational level of primary school and lower was significantly associated with non-adherence to ART. The pooled AOR of non-adherence to ART for patients with educational level of primary school and below was 3.52 (95%CI: 1.68, 7.40; I^2^ = 62.49%; P < 0.01) (Fig. 5).

**Fig. 6:**
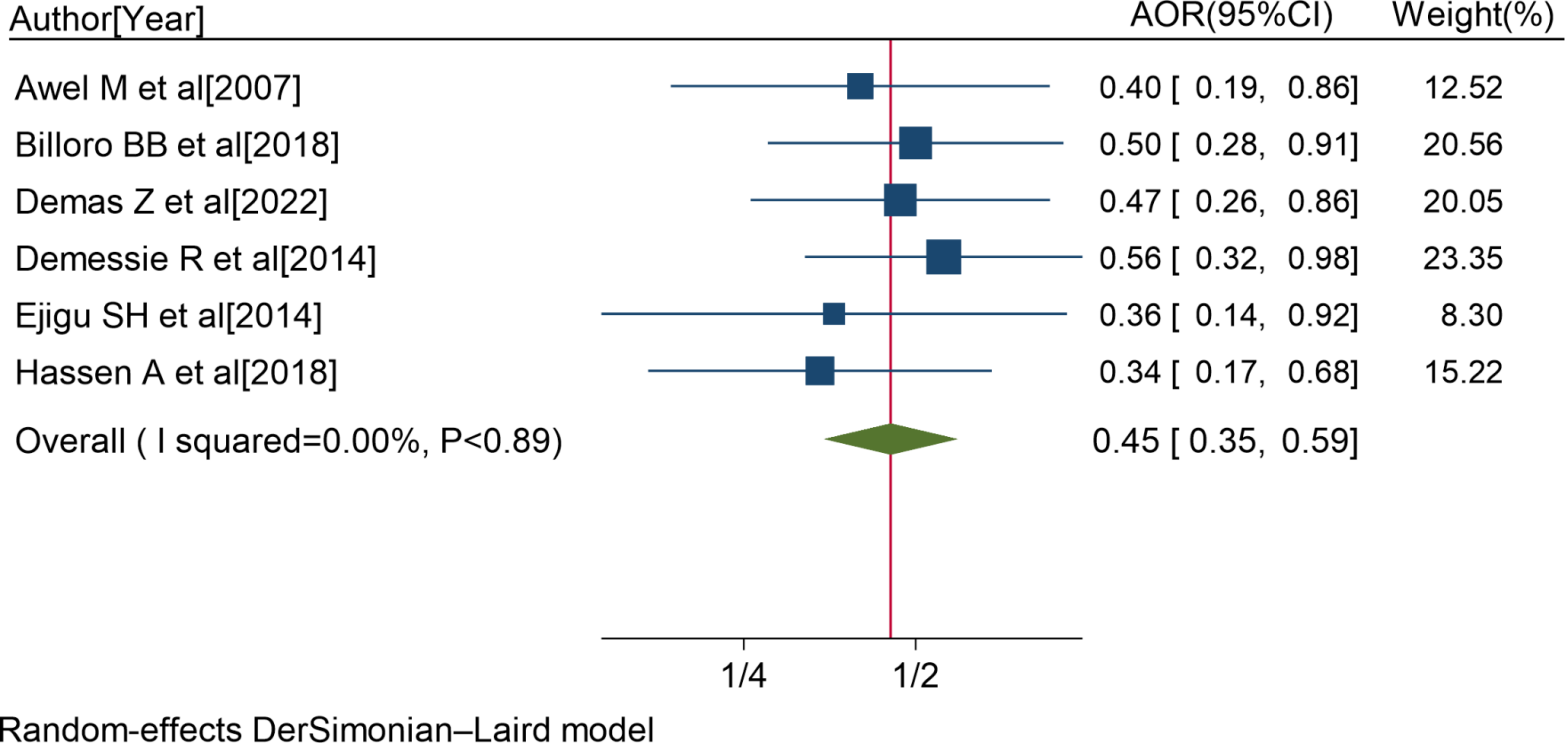
Forest plot of the adjusted odds ratios with corresponding 95% CIs of studies on the association of co-medications and non-adherence to ART. The midpoint and the length of each segment indicated an AOR and a 95% CI; and the diamond shape showed the combined AOR of all studies

Six studies [8, 18, 34, 57, 59, 64] showed a significant association between taking co-medications and non-adherence to ART. The pooled AOR of non-adherence to ART for patients with co-medications was 0.45 (95%CI: 0.35, 0.59; I^2^ = 0.00%; P < 0.89) (Fig. 6).

**Fig. 7:**
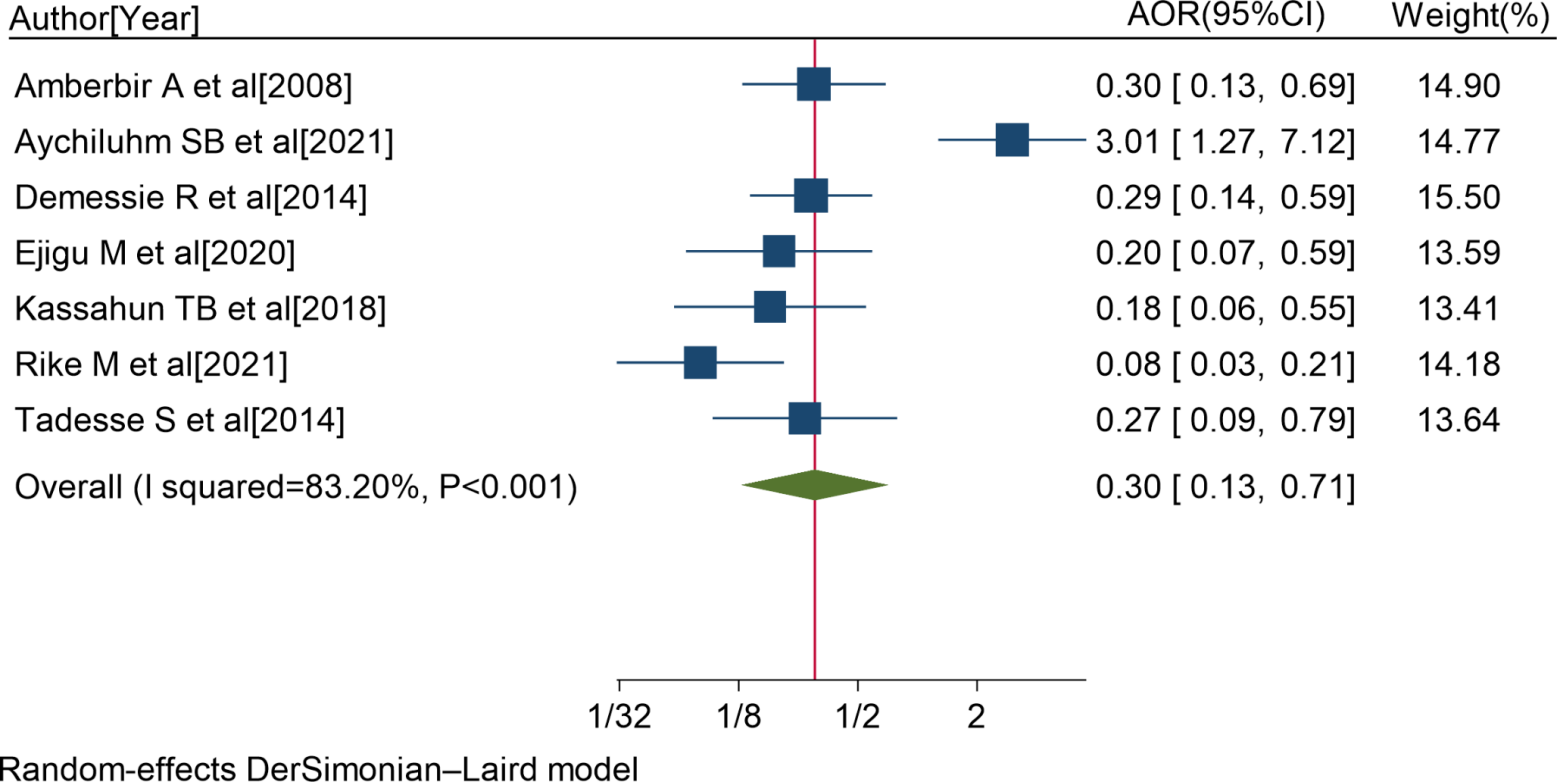
Forest plot of the adjusted odds ratios with corresponding 95% CIs of studies on the association of not using memory aids and non-adherence to ART. The midpoint and the length of each segment indicated an AOR and a 95% CI; and the diamond shape showed the combined AOR of all studies

Seven studies [6, 8, 27, 62, 72, 73, 79] also reported a significant association between not using memory aids and non-adherence to ART. The pooled AOR of non-adherence to ART for patients who were not using memory aids was 0.30 (95%CI: 0.13, 0.71; I^2^ = 83.20%; P < 0.001) (Fig. 7).

Six studies [19, 44, 53, 58, 60, 79] showed a significant association between depression and non-adherence to ART. The pooled AOR of non-adherence to ART for patients with depression was 2.0 (95%CI: 1.05, 3.79).

Three studies [5, 62, 83] reported a significant association between comorbidity and non-adherence to ART. The pooled AOR of non-adherence to ART for patients with comorbidity was 2.12 (95%CI: 1.16, 3.09).

Three studies [35, 69, 71] also showed a significant association between under-nutrition and non-adherence to ART. The pooled AOR of non-adherence to ART for patients with under-nutrition was 2.02 (95%CI: 1.20, 3.43).

Three studies [55, 57, 64] also showed a significant association between not believing on ART controls HIV and non-adherence to ART. The pooled AOR of non-adherence to ART for patients who didn’t belief that ART controls HIV was 2.31 (95%CI: 1.92, 2.77).

Two studies [34, 64] reported a significant association between lack of access to health facilities and non-adherence to ART. The pooled AOR of non-adherence to ART for patients with lack of access to health facilities was 3.86 (95%CI: 1.10, 13.51).

Two studies [1, 72] showed a significant association between taking ART pills uncomfortably while others looking and non-adherence to ART. The pooled AOR of non-adherence to ART for patients taking ART pills uncomfortably while others looking was 5.21 (95%CI: 2.56, 10.53).

Thus, PLWH with educational level of primary school and lower were 3.5 times more likely to have non-adherence to ART compared with those with educational level of secondary school and above.

Likewise, PLWH who were taking co-medications were 2.2 times more likely to develop non-adherence to ART than PLWH who didn’t take co-medications.

Those PLWH who were not using memory aids were also 3.3 times more likely to have non-adherence to ART compared to PLWH who were using memory aids.

In this study, adult PLWH with depression were 2 times more likely to develop non-adherence to ART than those who didn’t have depression.

Similarly, PLWH with comorbidity were 2.1 times more likely to develop non-adherence to ART than PLWH without comorbidity.

Besides, PLWH with under-nutrition (BMI < 18.5 Kg/m^2^) were 2 times more likely to have non-adherence to ART compared to those without under-nutrition.

PLWH who didn’t believe on ART can control HIV were 2.3 times more likely to have non-adherence to ART than those who believed that ART controls HIV.

Additionally, PLWH with lack of access to health facilities were 3.9 times more likely to develop non-adherence to ART than those who had access to health facilities. Moreover, PLWH who were taking ART pills uncomfortably while others looking were 5.2 times more likely to develop non-adherence to ART than their counterparts.

## Discussion

This review aimed to determine the overall pooled prevalence of non-adherence to ART and its predictors in Ethiopia. In this study, the overall pooled prevalence of non-adherence to ART was 20.68% (95% CI: 17.74, 23.61; I^2^ = 98.40%; p < 0.001), which was higher than the study findings conducted in India (12.4%) [84], Vietnam (11.5%) [85], Kenya (18%) [86], Indonesia (18.5%) [87], Orlu of Nigeria (17.1%) [14], Monze and Nyimba districts of Zambia (16.5%) [88], and Kasama district of Zambia (18%) [89].

But the finding was lower than the study findings conducted in Senegal (24.6%) [90], Northwest province of Zambia (27%) [91], China (28%) [92], Cameroon (37.78%) [7], Nepal (40%) [93], South Nigeria (40.1%) [94] and Benin city, Nigeria (41.9%) [95]. This discrepancy could be due to differences in study settings, methodologies, health care delivery systems across settings and the existence of socio-cultural variations [6].

Furthermore, the finding of this study reported that adult PLWH with educational level of primary school and lower were 3.5 times more likely to have non-adherence to ART than those with educational level of secondary school and above. This finding was consistent with the study finding conducted in Cameroon [7] and Vietnam [85]. It is possible that the low level of education among individuals also leads to a low level of awareness about HIV and its treatment. Consequently, the non-adherence to ART may be high [5].

According to the findings of this study, PLWH who were taking co-medications were also 2.2 times more likely to develop non-adherence to ART than PLWH who didn’t take co-medications. Patients who take co-medications may experience perceived side effects, which could lead them to skip or miss their ART regimens [96].

It has been found that not using memory aids is a significant predictor of non-adherence to ART. People living with HIV who do not use memory aids are 3.3 times more likely to have non-adherence to ART compared to those who do use memory aids. The finding was in line with a study conducted in Vietnam [97]. One way to make it easier for patients to remember when to take their medication is by using memory aids [98].

The finding of this study also showed that Adult PLWH with depression were 2 times more likely to have non-adherence to ART than those who didn’t have depression. This might be explained as patients become depressed, they would be hopeless and in turn, they might skip or miss to take their regular ART regimens. This finding supports the role of HIV/AIDS counselors in screening for depression and providing treatment when appropriate, either directly or through collaboration with the mental health professionals [53].

Similarly, the finding indicated that PLWH with comorbidity were 2.1 times more likely to develop non-adherence to ART than PLWH without comorbidity. The possible reason might be explained as patients with comorbid diseases could be tired and limit them from taking ART regimens regularly [5].

Besides, PLWH with under-nutrition were 2 times more likely to have non-adherence to ART compared to those without under-nutrition. This finding is consistent with studies conducted in Zambian and Uganda [99, 100]. Patients with low nutritional status could have reduced immunity, posing the patients to be suffered from frequent opportunistic infections. This can affect drug metabolism, absorption and efficacy of drugs, potentially leading to non-adherence to ART [101].

Similarly, PLWH who didn’t belief that ART can control the virus were 2.3 times more likely to be non-adherent to ART compared to those who believed ART can control the virus. It might be explained that this false belief could encourage patients to cease taking ART regimens [102].

Additionally, PLWH who don’t have access to health facilities are nearly four times more likely to struggle with adherence to antiretroviral therapy (ART) compared to those who do have access to health facilities. This finding is supported with a study conducted in Ghana [103]. PLWH who were willing to take ART could became non-adherent because of difficulties in reaching the treatment centers due to unexpected transport and other strikes; long travel distance; geographical difficulty including lack of transportation services in many remote areas; and the seasonal deterioration of poorer roads during the rainy season [104].

Moreover, PLWH who were taking ART pills uncomfortably while others looking were 5.2 times more likely to develop non-adherence to ART than their counterparts. This finding was congruent with a study finding conducted in Addis Ababa [105]. Patients may be uncomfortable while taking ART pills in front of others due to fear of stigma from disclosing their HIV status to the family, colleagues, and the community. This could lead to non-adherence to ART [106].

### Strength and Limitation of the Study

This review is the first study to combine the results of multiple studies conducted in Ethiopia, providing stronger evidence on non-adherence to ART and the factors predicting it. The study also included many study participants (n = 36,317) exceeding the sample sizes of the primary studies included. While all the studies are of good quality, it should be noted that the majority of the studies analyzed were cross-sectional. Moreover, the study couldn’t perform a sub-group analysis depending on the study setting and design.

## Conclusions

The overall pooled prevalence of non-adherence to ART was considerably high in Ethiopia. The review has revealed that primary school and lower, taking co-medications, not using memory aids, depression, comorbidity, under-nutrition, not believing on ART controls HIV, lacking access to health facilities and taking ART pills uncomfortably while others looking were independent predictors of non-adherence to ART in Ethiopia. Therefore, healthcare providers, adherence counselors and supporters should detect non-adherence behaviors and patients’ difficulties with ART early, and provide intensive counseling and manage the difficulties appropriately to promote adherence and improve the treatment outcome.

### Electronic supplementary material

Below is the link to the electronic supplementary material.

Supplementary Material 1

Supplementary Material 2

## Data Availability

All necessary data and supplementary materials were included in the manuscript.

## Abbreviations

AIDS: Acquired Immunodeficiency Syndrome
AOR: Adjusted Odds Ratio
ART: Anti-Retroviral Therapy
ARV: Anti-Retro Viral
BMI: Body Mass Index
CD4: Cluster of Differentiation 4
CI: Confidence Interval
EDHS: Ethiopian Demographic and Health Survey
HIV: Human Immunodeficiency Virus
JBI: Joanna Briggs Institute
OR: Odds Ratio
PLWH: People Living With HIV
PRISMA: Preferred Reporting Items for Systematic Reviews and Meta-Analyses
USA: United States of America
WHO: World Health Organization
