# Supplementary material for: Non-Adherence to Anti-Retroviral Therapy Among Adult People Living with HIV in Ethiopia: Systematic Review and Meta-Analysis
Source: AIDS Behav. 2023 Dec 29;28(2):609–24. doi: 10.1007/s10461-023-04252-4 (PMC10876791; doi:10.1007/s10461-023-04252-4)
Supplement: Supplementary file 1 — Supplementary Material 1 [file 10461_2023_4252_MOESM1_ESM.docx]

**Non-adherence to Anti-retroviral Therapy among Adult People Living with HIV in Ethiopia: A Systematic Review and Meta-Analysis**

**AIDS and Behavior**

Tigabu Munye Aytenew^1^*, Solomon Demis^2^, Binyam Minuye Birhane^8^, Worku Necho Asferie^2^, Habtamu Shimels^2^, Amare Simegn Ayele^3^, Gedefaye Nibret^3^, Amare Kassaw^4^, Sintayehu Asnakew^5^, Yohannes Tesfahun^6^, Henock Andualem^7^, Berihun Bantie^1^, Gebrie Kassaw^1^, Demewoz Kefale^4^, Shegaw Zeleke^1^

^1^Department of Nursing, College of Health Sciences, Debre Tabor University, Debre Tabor, Ethiopia

^2^Department of Maternity and Neonatal Nursing, College of Health Sciences, Debre Tabor University, Debre Tabor, Ethiopia

^3^Department of Midwifery, College of Health Sciences, Debre Tabor University, Debre Tabor, Ethiopia

^4^Department of Pediatrics and Child health Nursing, College of Health Sciences, Debre Tabor University, Debre Tabor, Ethiopia

^5^Department of Psychiatry, College of Health Sciences, Debre Tabor University, Debre Tabor, Ethiopia

^6^Department of Emergency and Critical Care Nursing, College of Health Sciences, Debre Tabor University, Debre Tabor, Ethiopia

^7^Department of Medical Laboratory, College of Health Sciences, Debre Tabor University, Debre Tabor, Ethiopia

^8^School of Public Health, University of Technology Sydney, Sydney, NSW, Australia

*Corresponding author: Tigabu Munye Aytenew*. Tele*: *+251921613861* Fax:

0581410533, and *Email:* [*tigabumunye21@gmail.com*](mailto:tigabumunye21@gmail.com)

SD: [solomondemis@gmail.com](mailto:solomondemis@gmail.com)

BM: [biniamminuye@yahoo.com](mailto:biniamminuye@yahoo.com)

WN: [workunecho@gmail.com](mailto:workunecho@gmail.com)

AS: [amaresimegn99@gmail.com](mailto:amaresimegn99@gmail.com)

GN: [gedefayen@gmail.com](mailto:gedefayen@gmail.com)

AK: [amarekassaw2009@gmail.com](mailto:amarekassaw2009@gmail.com)

SA: [sintie579@gmail.com](mailto:sintie579@gmail.com)

YT: [tesfahunyohannes08@gmail.com](mailto:tesfahunyohannes08@gmail.com)

HA: [henokyaa@yahoo.com](mailto:henokyaa@yahoo.com)

BB: [berihunbante@gmail.com](mailto:berihunbante@gmail.com)

GK: [gebriekassaw27@gmail.com](mailto:gebriekassaw27@gmail.com)

DK: [demewozk@yahoo.com](mailto:demewozk@yahoo.com)

SZ: [shegawzn@gmail.com](mailto:shegawzn@gmail.com)

Supplemental Table 1**:** PRISMA checklist

| **Section/Topic** | **#** | **Checklist item** | Reported on page # |
| --- | --- | --- | --- |
| **TITLE** |  |  |  |
| Title | 1 | Identify the report as a systematic review, meta-analysis, or both. | 1 |
| **ABSTRACT** | | |  |
| Abstract | 2 | Provide a structured summary including, as applicable: background; objectives; data sources; study eligibility criteria, participants, and interventions; study appraisal and synthesis methods; results; limitations; conclusions and implications of key findings; systematic review registration number. | 2 |
| **INTRODUCTION** | | |  |
| Rationale | 3 | Describe the rationale for the review in the context of existing knowledge. | 4-5 |
| Objectives | 4 | Provide an explicit statement of questions being addressed with reference to participants, interventions, comparisons, outcomes, and study design (PICOS). | 5 |
| **METHODS** | | |  |
| Protocol and registration | 5 | Indicate if a review protocol exists, if and where it can be accessed (e.g., Web address), and, if available, provide registration information including registration number. | 6 |
| Eligibility criteria | 6 | Specify study characteristics (e.g., PICOS, length of follow-up) and report characteristics (e.g., years considered, language, publication status) used as criteria for eligibility, giving rationale. | 6 |
| Information sources | 7 | Specify all databases, registers, websites, organisations, reference lists and other sources searched or consulted to identify studies. Specify the date when each source was last searched or consulted. | 6 |
| Search strategy | 8 | Present the full search strategies for all databases, registers and websites, including any filters and limits used. | 6 |
| Study selection | 9 | State the process for selecting studies (i.e., screening, eligibility, included in systematic review, and, if applicable, included in the meta-analysis). | 7 |
| Data collection process | 10 | Describe method of data extraction from reports (e.g., piloted forms, independently, in duplicate) and any processes for obtaining and confirming data from investigators. | 7 |
| Data items | 11 | List and define all variables for which data were sought (e.g., PICOS, funding sources) and any assumptions and simplifications made. | 7 |
| Risk of bias in individual studies | 12 | Describe methods used for assessing risk of bias of individual studies (including specification of whether this was done at the study or outcome level), and how this information is to be used in any data synthesis. | 14 |
| Summary measures | 13 | State the principal summary measure(s) (e.g., risk ratio, mean difference) used in the synthesis. | 10 |
| Synthesis of results | 14 | Describe the methods of handling data and combining results of studies, if done, including measures of consistency (e.g., I2) for each meta-analysis. | 7-9 |
| Risk of bias across studies | 15 | Specify any assessment of risk of bias that may affect the cumulative evidence (e.g., publication bias, selective reporting within studies) | 14 |
| Additional analyses | 16 | Describe methods of additional analyses (e.g., sensitivity or subgroup  analyses, meta-regression), if done, indicating which were pre-specified. | 14-17 |
| **RESULTS** | | |  |
| Study selection | 17 | Give numbers of studies screened, assessed for eligibility, and included in the review, with reasons for exclusions at each stage, ideally with a flow diagram. | 7 & Fig1 |
| Study characteristics | 18 | For each study, present characteristics for which data were extracted (e.g., study size, PICOS, follow-up period) and provide the citations. | 9 & Table1 |
| Risk of bias within studies | 19 | Present data on risk of bias of each included study and, if available, any outcome level assessment. | 14& S/Table2 |
| Results of individual studies | 20 | For all outcomes considered (benefits or harms), present, for each study: (a) simple summary data for each intervention group (b) effect estimates and confidence intervals, ideally with a forest plot. | 14 & Fig2 |
| Synthesis of results | 21 | Present results of each meta-analysis done, including confidence intervals and measures of consistency. | 7-15 |
| Reporting biases | 22 | Present results of any assessment of risk of bias across studies. | 14& Table5 |
| Additional analysis | 23 | Give results of additional analyses, if done (e.g., sensitivity or subgroup analyses, meta-regression. | 14&15 |
| **DISCUSSION** | | |  |
| Summary of evidence | 24 | Summarize the main findings including the strength of evidence for each main outcome; consider their relevance to key groups (e.g., healthcare providers, users, and policy makers). | 22-25 |
| Limitations | 25 | Discuss limitations at study and outcome level (e.g., risk of bias), and at review-level (e.g., incomplete retrieval of identified research, reporting bias). | 25 |
| Conclusions | 26 | Provide a general interpretation of the results in the context of other evidence, and implications for future research. | 26 |
| **OTHERS INFORMATION** | | |  |
| Funding | 27 | Describe sources of funding for the systematic review and other support (e.g., supply of data); role of funders for the systematic review. | 27 |
| Competing interests | 28 | Declare any competing interests of review authors. | 27 |
| Availability of data, code and other materials | 29 | Report which of the data are publicly available and where they can be found: template data collection forms; data extracted from included studies; data used for all analyses; analytic code; any other materials used in the review. | 27 |

Note: From: Page MJ, McKenzie JE, Bossuyt PM, Boutron I, Hoffmann TC, Mulrow CD, et al. The PRISMA 2020 statement: an updated guideline for reporting systematic reviews and Meta analyses. BMJ 2021;372: n71. doi: 10.1136/bmj. n71. For more information, visit: http://www.prisma-statement.org/
