# Supplementary material for: Non-Adherence to Anti-Retroviral Therapy Among Adult People Living with HIV in Ethiopia: Systematic Review and Meta-Analysis
Source: AIDS Behav. 2023 Dec 29;28(2):609–24. doi: 10.1007/s10461-023-04252-4 (PMC10876791; doi:10.1007/s10461-023-04252-4)
Supplement: Supplementary file 2 — Supplementary Material 2 [file 10461_2023_4252_MOESM2_ESM.docx]

**Non-adherence to Anti-retroviral Therapy among Adult People Living with HIV in Ethiopia: A Systematic Review and Meta-Analysis**

**AIDS and Behavior**

Tigabu Munye Aytenew^1^*, Solomon Demis^2^, Binyam Minuye Birhane^8^, Worku Necho Asferie^2^, Habtamu Shimels^2^, Amare Simegn Ayele^3^, Gedefaye Nibret^3^, Amare Kassaw^4^, Sintayehu Asnakew^5^, Yohannes Tesfahun^6^, Henock Andualem^7^, Berihun Bantie^1^, Gebrie Kassaw^1^, Demewoz Kefale^4^, Shegaw Zeleke^1^

^1^Department of Nursing, College of Health Sciences, Debre Tabor University, Debre Tabor, Ethiopia

^2^Department of Maternity and Neonatal Nursing, College of Health Sciences, Debre Tabor University, Debre Tabor, Ethiopia

^3^Department of Midwifery, College of Health Sciences, Debre Tabor University, Debre Tabor, Ethiopia

^4^Department of Pediatrics and Child health Nursing, College of Health Sciences, Debre Tabor University, Debre Tabor, Ethiopia

^5^Department of Psychiatry, College of Health Sciences, Debre Tabor University, Debre Tabor, Ethiopia

^6^Department of Emergency and Critical Care Nursing, College of Health Sciences, Debre Tabor University, Debre Tabor, Ethiopia

^7^Department of Medical Laboratory, College of Health Sciences, Debre Tabor University, Debre Tabor, Ethiopia

^8^School of Public Health, University of Technology Sydney, Sydney, NSW, Australia

*Corresponding author: Tigabu Munye Aytenew*. Tele*: *+251921613861* Fax:

0581410533, and *Email:* [*tigabumunye21@gmail.com*](mailto:tigabumunye21@gmail.com)

SD: [solomondemis@gmail.com](mailto:solomondemis@gmail.com)

BM: [biniamminuye@yahoo.com](mailto:biniamminuye@yahoo.com)

WN: [workunecho@gmail.com](mailto:workunecho@gmail.com)

AS: [amaresimegn99@gmail.com](mailto:amaresimegn99@gmail.com)

GN: [gedefayen@gmail.com](mailto:gedefayen@gmail.com)

AK: [amarekassaw2009@gmail.com](mailto:amarekassaw2009@gmail.com)

SA: [sintie579@gmail.com](mailto:sintie579@gmail.com)

YT: [tesfahunyohannes08@gmail.com](mailto:tesfahunyohannes08@gmail.com)

HA: [henokyaa@yahoo.com](mailto:henokyaa@yahoo.com)

BB: [berihunbante@gmail.com](mailto:berihunbante@gmail.com)

GK: [gebriekassaw27@gmail.com](mailto:gebriekassaw27@gmail.com)

DK: [demewozk@yahoo.com](mailto:demewozk@yahoo.com)

SZ: [shegawzn@gmail.com](mailto:shegawzn@gmail.com)

**Supplemental Table 2: Quality assessment of the included studies using the Joanna Briggs Institute (JBI) quality appraisal criteria**

| 1. **For cross-sectional studies** | | | | | | | | | | | | | | | | | |  |
| --- | --- | --- | --- | --- | --- | --- | --- | --- | --- | --- | --- | --- | --- | --- | --- | --- | --- | --- |
| **S/N** | **Author [Year]** | | **Criteria** | | | | | | | | | | | **Scores** | | | **Overall quality** |  |
|  |  |  | Clearly defined inclusion criteria | Describing the study settings participants | Valid &reliable exposure measurement | Objective &standard criteria for measurement | Identified confounder | | Strategies to deal with confounder | Valid & reliable outcome measurement | | Appropriate statistical analysis |  | | |  | |  |
|  | Abera A et al | | Y | Y | Y | Y | N | | Y | Y | | Y | 7 | | | Low risk | |  |
|  | Alagaw A et al | | Y | Y | Y | Y | N | | Y | Y | | Y | 7 | | | Low risk | |  |
|  | Angelo AT et al | | Y | Y | Y | Y | N | | Y | Y | | Y | 7 | | | Low risk | |  |
|  | Assefa N *et al* | | N | Y | Y | Y | N | | Y | N | | Y | 5 | | | Low risk | |  |
|  | Awel M et al | | Y | Y | Y | Y | N | | Y | Y | | Y | 7 | | | Low risk | |  |
|  | Aychiluhm SB et | | N | Y | Y | Y | N | | Y | Y | | Y | 6 | | | Low risk | |  |
|  | Beyene KA et al | | Y | Y | Y | Y | N | | Y | Y | | Y | 7 | | | Low risk | |  |
|  | Billoro BB *et al* | | N | Y | Y | Y | N | | Y | Y | | N | 5 | | | Low risk | |  |
|  | Chaka TE *et al* | | Y | Y | Y | Y | N | | Y | Y | | Y | 7 | | | Low risk | |  |
|  | Debito T *et al* | | Y | Y | Y | Y | N | | Y | Y | | Y | 7 | | | Low risk | |  |
|  | Demas Z *et al* | | Y | Y | Y | Y | N | | Y | Y | | Y | 7 | | | Low risk | |  |
|  | Demeke B *et al* | | Y | Y | Y | Y | N | | Y | Y | | Y | 7 | | | Low risk | |  |
|  | Demessie R *et a* | | Y | Y | Y | N | N | | Y | Y | | Y | 6 | | | Low risk | |  |
|  | Dibaba D *et al* | | Y | Y | Y | Y | N | | Y | Y | | Y | 7 | | | Low risk | |  |
|  | Ejigu M *et al* | | Y | Y | Y | Y | N | | Y | Y | | Y | 7 | | | Low risk | |  |
|  | Ejigu SH *et a*l | | Y | Y | Y | Y | N | | Y | Y | | Y | 7 | | | Low risk | |  |
|  | G/egziabher TT | | Y | Y | Y | Y | N | | Y | Y | | Y | 7 | | | Low risk | |  |
|  | Hassen A *et al* | | Y | Y | Y | Y | N | | Y | Y | | Y | 7 | | | Low risk | |  |
|  | Hebo SH *et al* | | Y | Y | Y | Y | N | | Y | Y | | Y | 7 | | | Low risk | |  |
|  | Hussen HS *et al* | | Y | Y | Y | Y | N | | Y | Y | | Y | 7 | | | Low risk | |  |
|  | Jima F *et al* | | Y | Y | Y | Y | N | | Y | Y | | Y | 7 | | | Low risk | |  |
|  | Kassahun TB et | | N | Y | Y | Y | N | | Y | Y | | Y | 6 | | | Low risk | |  |
|  | Koyra HC *et al* | | Y | Y | Y | Y | N | | Y | Y | | Y | 7 | | | Low risk | |  |
|  | Letta S *et al* | | Y | Y | Y | Y | N | | Y | Y | | Y | 7 | | | Low risk | |  |
|  | Markos E *et al* | | Y | Y | Y | Y | N | | Y | Y | | Y | 7 | | | Low risk | |  |
|  | Mengistie A *et al* | | Y | Y | Y | Y | N | | Y | Y | | Y | 7 | | | Low risk | |  |
|  | Mitku H *et al* | | Y | Y | Y | Y | N | | Y | Y | | Y | 7 | | | Low risk | |  |
|  | Mitku AA *et al* | | Y | Y | Y | Y | N | | Y | Y | | Y | 7 | | | Low risk | |  |
|  | Mohammed M *e* | | Y | N | Y | Y | N | | Y | N | | Y | 5 | | | Low risk | |  |
|  | Molla AA *et al* | | Y | Y | Y | Y | N | | Y | Y | | Y | 7 | | | Low risk | |  |
|  | Negash E *et al* | | Y | Y | Y | Y | N | | Y | Y | | Y | 7 | | | Low risk | |  |
|  | Nigusso FT *et al* | | N | Y | Y | Y | N | | Y | Y | | Y | 6 | | | Low risk | |  |
|  | Reta H *et al* | | Y | Y | Y | Y | N | | Y | Y | | Y | 7 | | | Low risk | |  |
|  | Rike M *et al* | | Y | Y | Y | Y | N | | Y | Y | | Y | 7 | | | Low risk | |  |
|  | Tadesse S *et al* | | Y | Y | Y | Y | N | | Y | Y | | Y | 7 | | | Low risk | |  |
|  | Tesfay S *et al* | | Y | Y | Y | Y | N | | Y | Y | | Y | 7 | | | Low risk | |  |
|  | Tessema B *et al* | | Y | Y | Y | Y | N | | Y | Y | | Y | 7 | | | Low risk | |  |
|  | Tiyou A *et al* | | Y | Y | Y | Y | N | | Y | Y | | Y | 7 | | | Low risk | |  |
|  | Tsega B *et al* | | N | Y | Y | Y | N | | Y | Y | | Y | 6 | | | Low risk | |  |
|  | Yadeta AD *et al* | | Y | Y | Y | Y | N | | Y | Y | | Y | 7 | | | Low risk | |  |
|  | Zeleke AB *et al* | | Y | Y | Y | Y | N | | Y | Y | | Y | 7 | | | Low risk | |  |
|  | Zewude SB *et al* | | Y | Y | Y | Y | N | | Y | Y | | Y | 7 | | | Low risk | |  |
|  | 1. **For cohort and case-control studies** | | | | | | | | | | | | | | | | | |
|  | **S/N** | | **Criteria** | | | | | | Amberbir A et al | | | Desta AA et | | | | Desta AA *et al* | | |
|  | 1. 1. | | Two groups are similar and recruited from the same population | | | | | | Y | | | Y | | | |  | | |
| 1. 2. | | Similar measurement of exposure both for exposed and unexposed groups | | | | | | Y | | | Y | | | | Y | | |  |
| 1. 3. | | Valid and reliable measurement of exposure | | | | | | Y | | | Y | | | | Y | | |  |
| - 1. 4 | | Identifying confounders | | | | | | N | | | N | | | | N | | |  |
| 1. 5. | | Strategies to deal with confounders | | | | | | N | | | N | | | | N | | |  |
| 1. 6. | | Groups are free of the outcomes at the beginning | | | | | | Y | | | Y | | | | Y | | |  |
| 1. 7. | | Valid and reliable measurement of outcomes | | | | | | Y | | | Y | | | | Y | | |  |
| 1. 8. | | Long enough follow-up time for the occurrence of outcomes | | | | | | Y | | | N | | | | Y | | |  |
| 1. 9. | | Complete follow-up time | | | | | | Y | | | Y | | | | Y | | |  |
| 1. 10. | | Strategies to address lost follow-up | | | | | | Y | | | Y | | | | Y | | |  |
| ***Percentage (%) of ʺYesʺ*** | | | | | | | | *8/10=80%* | | | *7/10=70%* | | | | *8/10=80%* | | |  |

*Note: Y, yes; N, No*

**Supplemental Table 3: Risk of bias assessment of the included studies**

| **S/N** | **Author [Year]** | **Criteria** | | | | | | | | | | **Scores** | **Overall risk of bias** |
| --- | --- | --- | --- | --- | --- | --- | --- | --- | --- | --- | --- | --- | --- |
|  |  | **External validity** | | | | **Internal validity** | | | | | |  |  |
|  |  | **Q1** | **Q2** | **Q3** | **Q4** | **Q5** | **Q6** | **Q7** | **Q8** | **Q9** | **Q10** |  |  |
|  | Abera A et al | Y | Y | N | Y | Y | Y | N | Y | Y | Y | 8 | Low risk |
|  | Alagaw A et al | Y | Y | Y | Y | Y | N | N | Y | N | Y | 7 | Low risk |
|  | Angelo AT et al | Y | Y | N | Y | N | Y | Y | Y | Y | Y | 8 | Low risk |
|  | Assefa N *et al* | Y | Y | N | Y | Y | Y | N | Y | Y | Y | 8 | Low risk |
|  | Awel M et al | Y | Y | N | Y | Y | Y | N | Y | N | Y | 7 | Low risk |
|  | Aychiluhm SB et | Y | Y | Y | Y | N | Y | N | Y | Y | Y | 8 | Low risk |
|  | Beyene KA et al | Y | Y | N | Y | Y | N | Y | Y | Y | Y | 8 | Low risk |
|  | Billoro BB *et al* | Y | Y | N | Y | Y | Y | N | Y | N | Y | 7 | Low risk |
|  | Chaka TE *et al* | Y | Y | Y | Y | N | Y | Y | Y | Y | Y | 8 | Low risk |
|  | Debito T *et al* | Y | Y | N | Y | Y | Y | N | Y | Y | Y | 8 | Low risk |
|  | Demas Z *et al* | Y | Y | N | Y | Y | Y | N | Y | Y | Y | 8 | Low risk |
|  | Demeke B *et al* | Y | Y | N | Y | Y | Y | N | Y | Y | Y | 8 | Low risk |
|  | Demessie R *et a* | N | Y | Y | Y | Y | N | Y | Y | Y | Y | 8 | Low risk |
|  | Dibaba D *et al* | Y | Y | N | Y | Y | Y | N | Y | Y | Y | 8 | Low risk |
|  | Ejigu M *et al* | Y | Y | N | Y | Y | Y | Y | N | Y | Y | 8 | Low risk |
|  | Ejigu SH *et a*l | Y | Y | N | Y | Y | Y | N | Y | Y | Y | 8 | Low risk |
|  | G/egziabher TT | Y | Y | N | Y | Y | Y | N | Y | Y | Y | 8 | Low risk |
|  | Hassen A *et al* | Y | Y | Y | Y | Y | N | Y | Y | N | Y | 8 | Low risk |
|  | Hebo SH *et al* | Y | Y | Y | N | Y | N | N | Y | Y | Y | 7 | Low risk |
|  | Hussen HS *et al* | Y | Y | N | Y | Y | Y | N | Y | Y | Y | 8 | Low risk |
|  | Jima F *et al* | Y | Y | N | Y | Y | N | Y | Y | Y | Y | 8 | Low risk |
|  | Kassahun TB et | Y | Y | N | Y | Y | Y | N | Y | Y | Y | 8 | Low risk |
|  | Koyra HC *et al* | Y | Y | N | Y | Y | Y | N | Y | Y | Y | 8 | Low risk |
|  | Letta S *et al* | Y | Y | Y | N | Y | N | Y | Y | Y | Y | 8 | Low risk |
|  | Markos E *et al* | Y | Y | N | Y | Y | Y | N | Y | Y | Y | 8 | Low risk |
|  | Mengistie A *et al* | Y | Y | N | Y | Y | Y | N | Y | Y | Y | 8 | Low risk |
|  | Mitku H *et al* | Y | Y | N | Y | Y | Y | N | Y | Y | Y | 8 | Low risk |
|  | Mitku AA *et al* | Y | Y | N | Y | Y | N | Y | Y | Y | Y | 8 | Low risk |
|  | Mohammed M *e* | Y | Y | Y | N | Y | Y | N | Y | Y | Y | 8 | Low risk |
|  | Molla AA *et al* | Y | Y | N | Y | Y | Y | N | Y | Y | Y | 8 | Low risk |
|  | Negash E *et al* | Y | Y | N | Y | Y | Y | N | Y | Y | Y | 8 | Low risk |
|  | Nigusso FT *et al* | Y | Y | N | Y | Y | N | Y | Y | Y | Y | 8 | Low risk |
|  | Reta H *et al* | N | Y | N | Y | Y | Y | N | Y | Y | Y | 7 | Low risk |
|  | Rike M *et al* | Y | Y | N | Y | Y | Y | N | Y | Y | Y | 8 | Low risk |
|  | Tadesse S *et al* | Y | Y | N | Y | Y | Y | N | Y | Y | Y | 8 | Low risk |
|  | Tesfay S *et al* | Y | Y | Y | N | Y | Y | N | Y | Y | Y | 8 | Low risk |
|  | Tessema B *et al* | Y | Y | N | Y | Y | N | Y | Y | Y | Y | 8 | Low risk |
|  | Tiyou A *et al* | Y | Y | N | Y | Y | Y | N | Y | Y | Y | 8 | Low risk |
|  | Tsega B *et al* | Y | Y | N | N | Y | Y | N | Y | Y | Y | 7 | Low risk |
|  | Yadeta AD *et al* | Y | Y | N | Y | Y | Y | N | Y | Y | Y | 8 | Low risk |
|  | Zeleke AB *et al* | Y | Y | N | Y | Y | Y | N | Y | Y | Y | 8 | Low risk |
|  | Zewude SB *et al* | Y | Y | N | Y | Y | Y | N | Y | Y | Y | 8 | Low risk |
|  | Amberbir A et al | Y | Y | Y | N | Y | Y | N | Y | Y | Y | 8 | Low risk |
|  | Desta AA et | Y | Y | N | Y | Y | N | Y | Y | Y | Y | 8 | Low risk |
|  | Desta AA *et al* | Y | Y | N | Y | Y | Y | N | Y | Y | Y | 8 | Low risk |

Note: Y, Yes; N, No; Q1, Representatives of the target population; Q2, Representativeness of the sampling frame; Q3, Random sampling or census; Q4, Minimal response bias; Q5, Data were collected directly; Q6, Acceptable case definition used in the study; Q7, Valid and reliable measurement; Q8, The same mode of data collection for all study subject; Q9, Appropriate length of prevalence period for parameter of interest and Q10, Appropriate numerators and denominators of interest.
